# Dynamic Tensile Stress-Compressive Stress Behavior of Thermoplastic Matrix Composite Materials Reinforced with Continuous Fiber for Automotive Damping and Anti-Vibration Structural Elements

**DOI:** 10.3390/ma13010005

**Published:** 2019-12-18

**Authors:** D. Tobalina-Baldeon, F. Sanz-Adan, M.A. Martinez-Calvo, J. Santamaría-Pena

**Affiliations:** Mechanical Engineering Department, Universidad de La Rioja, 26004 Logroño, Spaintachinto@gmail.com (J.S.-P.)

**Keywords:** continuous fiber thermoplastic, CFRTP, rubber-composite, composite’s dynamic property, damping system, vulcanized rubber, automotive

## Abstract

Continuous Fibers-Reinforced Thermoplastic Composites (CFRTP) are presented as light materials, capable of offering a short production time with the possibility of being recycled. These properties make them ideal for automotive applications, aiming to reduce the consumption and emission of polluting gases. This article analyzed the dynamic tensile stress-compressive stress behavior of CFRTP in structural elements of the car with anti-vibration and damping functions. The data available in the literature on the reliable and usual compliance of the properties required for CFRTP, to be applied in the automotive structural elements, is scarce and insufficient. In order to analyze whether CFRTP feeds the demanding requirements of car manufacturers and if they provide advantages over the metal materials currently used, this article developed a method of reliable verification of their dynamic tensile and compression behavior. The methodology developed could be used as a guide to characterizing any combination of vulcanized rubber adhesive joints with CFRTP, regardless of the materials and additives used. The results obtained showed that there exists CFRTP that fits the requirements of the car manufacturers for this type of component and also offers dynamic advantages over the materials currently used as anti-vibration elements.

## 1. Introduction

The constant increase in vehicle production, along with the increase in the industry in recent decades, results in historical maximums in CO_2eq_ levels, reaching 415 ppm in May 2019. This historical maximum has led to one of the main objectives of the current society; the decarbonization of road transport since “only passenger vehicles are responsible for 12% of CO_2eq_ emissions” [1].

The EU regulation requires “reducing CO_2eq_ emissions from cars to a maximum of 130 g/km for those manufactured from 2015 and 95 g/km by 2021” [2]. This measure represents a 40% reduction compared to 2007. To achieve the objectives defined at the 2015 Paris Climate Conference (COP21), “The EU will aim at reaching an ambitious, legally-binding and dynamic agreement, with the objective of keeping global warming below 2 °C. In order to achieve this objective, the Council stressed that global greenhouse gas emissions need to peak by 2020 at the latest, be reduced by at least 50% by 2050” [3].

China aims to reduce up to 107 g/km by 2020 and USA 105 g/km by 2020.

These objectives, willing to help reducing climate change, will mean annual savings for the consumer of €600 according to the “target” of 2025 and up to €1500 per year for cars purchased in 2030 [2].

There are different lines of research that seek to reduce CO_2eq_ emissions: efficient propulsion system, aerodynamic improvement, rolling resistance reduction, weight reduction, hybrid car, electric car [4].

Weight reduction is the research line that has more projection since it reduces the consumption of fossil fuel vehicles and increases the autonomy of hybrid and electric vehicles. If a mid-range car (1.2 to 1.5 tm) gets 100 kg moved, fuel consumption decreases between 0.3 and 0.6 L/km, and CO_2eq_ emissions are reduced by approximately 10 g/km.

**Figure 1:**
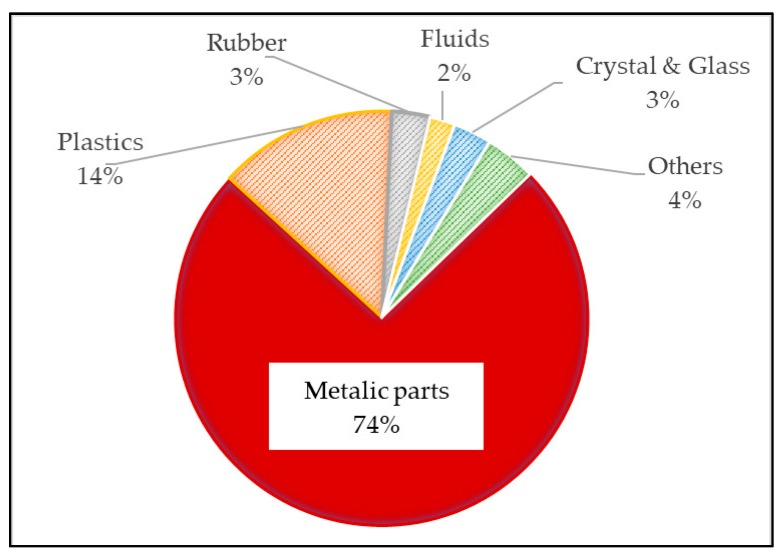
Materials used in passenger cars.

The steel is currently the main material present in the structure of vehicles (Figure 1). It is estimated that using high-performance special steels could lighten a car around 50/70 kg. When using special aluminum, the estimated maximum reduction would be 150 kg. However, with the use of composites, more than 200 kg could be lightened.

**Figure 2:**
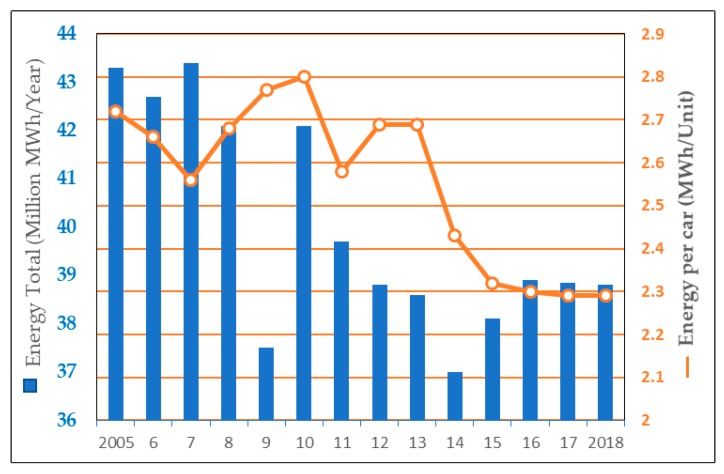
Energy consumption in vehicle production. Evolution 2005 to 2018.

The lightness of plastics, in addition to weight reduction, allows systems and components to be made more sophisticated and cheaper than with steels. In Figure 2, provided by the European Association of Automobile Manufacturers (A) [5], it can be seen that—thanks to the implantation of plastics—the energy consumed during its manufacture is lower.

In order to continue reducing the weight, it is necessary to investigate and develop components with lightweight materials and high performance that can be used in structural elements capable of complying with the increasing mechanical requirements and with the sustainability of road transport (reduction of fuel consumption, increase of the life cycle of the materials used, and short production times capable of satisfying the demand of the automotive sector).

Currently, the most commonly used thermoplastic materials are reinforced with staple fibers, such as short fiber or glass balls, mainly [6,7,8]. However, there is great potential to develop the use of high-performance continuous fiber-reinforced thermoplastics.

**Figure 3:**
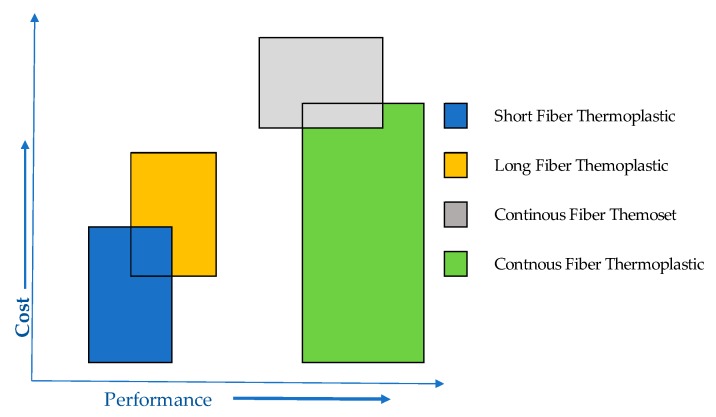
Relation cost/performance of composite materials.

The most used composites are those of polymer matrix of Thermoplastic structure Reinforced with continuous Fiber-glass or Fiber-carbon (CFRTP), which are light with stable mechanical properties and transformable in automated industrial processes [9]. In addition to being recyclable [10], they contribute to comply with EU Directive 2000/53/EC: “the total percentage of preparation for reuse and recycling will be at least 85% of the average weight per vehicle and year”. Figure 3 shows its cost and performance evolution with respect to other composite materials.

Nishida [11] compared “the mechanical features of thermoplastic and thermoset epoxy carbon textile composites. As the main outcome, the composite with highly polymerized thermoplastic epoxy has better mechanical performance than the conventional thermoset epoxy textile composite”.

The importance of these new materials is such that the European Union promoted the “SEAM cluster (October 2012–September 2016)” to carry out four R & D projects [12], aiming to develop a light electric car through the use of reinforced polymers with fiber:

The ALIVE project [13] focused on developing a light electric vehicle using fiber-reinforced composites.

The first works on the damping analysis of fiber-reinforced composite materials were exhibited by Gibson and Plunket [14] and Gibson and Wilson [15] in the late 1970s. In 1973, Adams and Bacon [16] presented a theoretical analysis on damping, in which they decomposed the dissipation energy of the composite into energy dissipations associated with the stresses of each component individually.

This work was further refined by Ni and Adams [17]. In the same line of theoretical analysis, dynamic calculation analysis was also developed by Lin et al. [18] and by Maheri and Adams [19].

Most of these studies are theoretical and are generally based on macroscopic analysis using the theory of classical lamination [20], based on the Kirchoff hypothesis, in which transverse deformations in the thickness direction are always neglected.

Atsushi Hosoi [21] “predicts transverse crack initiation in Carbon Fiber-Reinforced Polymer Composites (CFRP) cross-ply and quasi-isotropic laminates under cyclic loading in the present study”. Analytical results are in agreement with experimental results obtained, but only studies thermoset materials.

This analysis tends to focus on unidirectional composite materials [22,23] and the theoretical study of the influence of fiber angle on these unidirectional materials [24,25]. Regarding the behavior of reinforced sheets at 90°, there are very few publications, and those that exist have generalized theoretical calculations yet to be refined [26,27,28].

It is, therefore, necessary to obtain experimental results of the new CFRTP materials available in the market. In this way, the dynamic behavior of the different two-way reinforced CFRTP composites can be known experimentally at 90°, obtaining real results when they are subjected to the same loads than the steel and aluminum vibration insulators structural components.

Tobalina and Sanz-Adan [29,30] characterized these materials through standardized tests for continuous fiber composite materials and compared them with the values provided by the CFRTP manufacturers (tensile strength, tensile modulus, flexural strength, and flexural modulus, such as can be seen in Table 1), and showed that the mechanical properties tested were superior to aluminum and most steels. Therefore, it is possible to obtain a weight reduction of 6–7 times with respect to steel and 2 times with respect to aluminum, that the carbon fiber always reaches load values higher than those of fiberglass, and that its behavior is always linear until it reaches a fragile break under any tensile stress.

In this study, the benefits and improvements that the use of CFRTPs would provide for automotive damping and anti-vibration structural elements against the commonly used steel were analyzed, to reduce weight and contribute to the reduction of CO_2equiv_ emissions.

To achieve this objective, a reliable verification method of their dynamic tensile and compression behavior was developed.

## 2. Materials and Methods

The properties that manufacturers have on the different varieties of thermoplastic composites depend on multiple factors, such as the percentage of fiber, the direction of the fibers, and the load applied, which were also made on standard test specimens of virgin material without thermoforming.

Therefore, these properties helped us to get an initial and general idea about the possible behavior of the material, but not all the properties are characterized, and their behavior changes after being shaped.

In Table 1, we could see the properties provided by one of the manufacturers (Bond-laminates, Brito, North Rhine-Westfalia, Germany) on their materials [Tepex ©]:

The mechanical properties, demonstrated in the tests carried out in thermoplastic matrix composites reinforced with continuous fiber, encouraged us to analyze their feasibility in the use of automotive structural elements for damping and anti-vibration purposes.

The possibility of replacing steel sheets with composite sheets would greatly lighten the damping and anti-vibration components of the vehicle.

**Figure 4:**
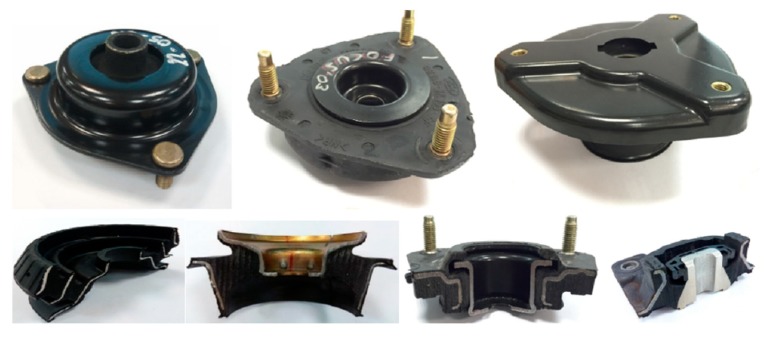
Automotive anti-vibration products: vulcanized rubber and steel.

If the reliability and repeatability of these properties are confirmed, these types of composites would be ideal for anti-vibration and damping structural elements. In Figure 4, some current anti-vibration systems composed of vulcanized rubber and steel are shown.

To certify compliance with these properties, manufacturers require dynamic tests on finished products, stiffness, and damping angle (“loss angle” or “damping angle”). Hydraulic parts (with liquid elements inside) or with anti-displacement adherent properties, which can produce noise during fatigue, sometimes also require specific noise studies.

In order to avoid vibrations or noise in vehicles, it is necessary to focus on dynamic properties and simplify their appearance in Equation (1):

*Observed vibrations (Response) = Force/Dynamic stiffness (restriction)* (1)

**Figure 5:**
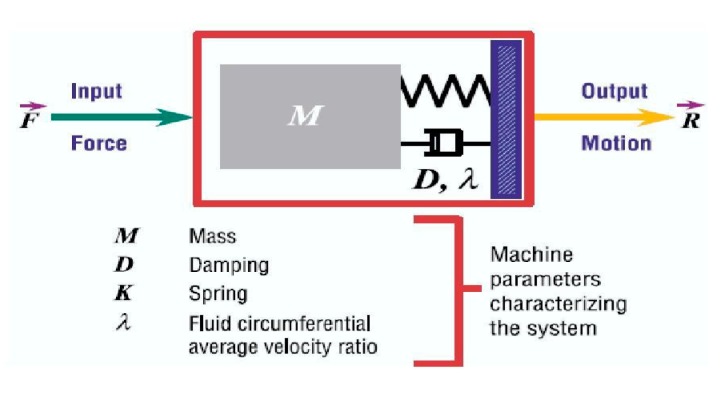
Dynamic system scheme.

If the force in Figure 5 is a constant preload, the spring would slowly compress, and the system would be in a new position. This static response is controlled only by the static spring stiffness (*K*).

A dynamic force changes in magnitude or direction over time. A dynamic input force will cause a dynamic output movement. The force (*F*) and the response (*R*) are vectors and have both magnitude and direction. Dynamic stiffness is the static stiffness of the system complemented by the effects of mass and damping.

The dynamic loss angle or “loss damping angle” represents the difference between stress and deformation, whose tangent is the loss factor. This value allows us to know the damping capacity of the material or part. The greater the angle of loss, the greater the damping capacity of the material.

In the family of automotive products, both dynamic stiffness and the angle of damping or loss are very important, as it is one of the main functional objectives and, therefore, one of the main reasons to use them.

In the vast majority of this type of parts (“silent blocks, stabilizer bushings, gear mounts”, Figure 4), the dynamic function is the responsibility of the rubber, which is the component of the piece responsible for absorbing vibrations and impacts, and it offers the dynamic properties required to this type of pieces.

**Figure 6:**
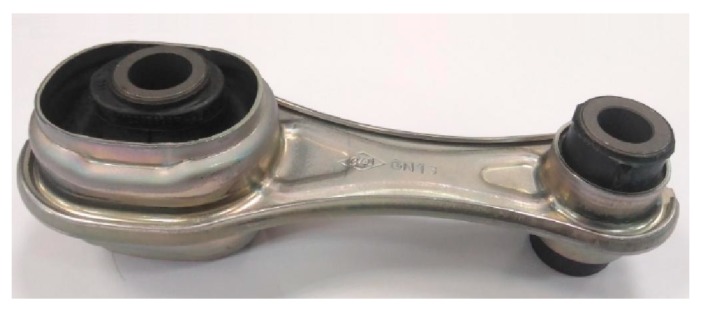
“Torque restrictor”—automotive anti-vibration structural element.

There are automotive components, such as “torque restrictors” (see Figure 6), in which the rigid part of the piece adds great value to its dynamic behavior. This type of parts generally work with tension stress-compression stress or torsion and, given their geometry, it is easy to replace steel or aluminum with CFRTP, in case it provides better performance. Therefore, if the dynamic results of the CFRTP are positive, their use would provide a great added value in the dynamic behavior that would result in a car with greater comfort and lower consumption, without sacrificing safety or recycling.

This analysis was based on a dynamic tensile stress-compression test on a rigid material test specimen (steel and CFRTP). Both steel and CFRTP test specimens were obtained directly from the respective suppliers.

The materials analyzed were CFRTP PA6 reinforced with 45% fiberglass (Composite 1), CFRTP PA66 reinforced with 45% carbon fiber (Composite 2), and C45 steel (ASTM A29 [31] and EN 10083-2 [32]), a material commonly used in this type of pieces, (two specimens, A1 and A2).

**Figure 7:**
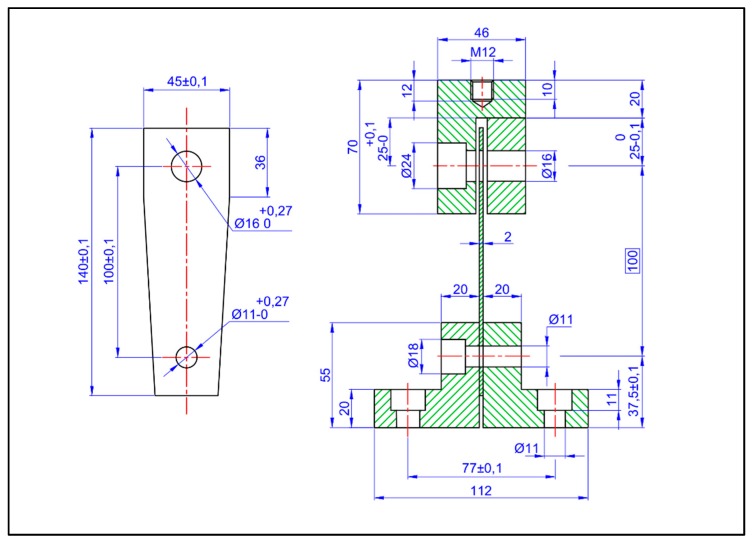
Dynamic tensile stress-compression stress test configuration.

The test specimens were the trapezoidal type with two holes at its ends. This design aroused from a standardized test tube concept so that it would not add uncertainties to the results of the properties to be analyzed. The dimensions are shown in Figure 7.

A fixing tool was manufactured, specifically designed for this test, to avoid any type of transmission of vibrations or intrusions in the dynamic results by the tool itself.

As the objective of this test was to characterize the dynamic properties of the CFRTP, the largest spectrum allowed by the available machine was analyzed, subjecting the specimens to a frequency scan from 10 to 300 Hz with 10 Hz intervals.

Two different cases were studied, with cycle amplitudes of ±0.05 mm and ±0.1 mm, for each of the frequency sweep values, coinciding with those required by vehicle manufacturers.

The defined frequency and amplitude conditions were applied to each specimen and the values of dynamic force. Dynamic stiffness and dynamic loss angle were collected.

The test was carried out at the facilities of the technical center of “*CMP Automotive Group*”, and the dynamic testing machine used was from the manufacturer “*Schenck*”.

The international standards that develop the test method and define the parameters, dimensions, and geometry of the test specimens are:

– ASTM D3039/D3039M—08 “Standard Test Method for Tensile Properties of Polymer Matrix Composite Materials” [33].
– ISO 527-4: 1997—“Plastics-Determination of tensile properties—Part 4: Test conditions for isotropic and orthotropic fiber-reinforced plastic composites. And its transposition to the European Union and the States that compose it, e.g., Spain (UNE-EN-ISO 527-4)” [34].

This part of the ISO 527 standard, belonging to the family of plastics standards, specifies the test conditions for the determination of the elastic limit, the deformation, the permissible loads, and the type of breakage of the materials of plastic composite, isotropic or orthotropic, fiber-reinforced when subjected to tensile stress. This method is suitable for the following materials:

- “Thermoplastic and thermosetting compounds reinforced with fibers that incorporate non-unidirectional reinforcements such as felts, fabrics (flat or windings), cut threads, combinations of these reinforcements, hybrids, windings, short or ground fibers or pre-impregnated materials (“prepregs”), using test pieces molded directly by injection” (ISO 527-1: 1993) [35].
- “Combinations of the above with unidirectional or multidirectional reinforcements, constructed from unidirectional layers, provided that such laminates are symmetrical for materials with full or mainly unidirectional reinforcements” (ISO 527-5) [36].
- Reinforced fibers, fiberglass, carbon, aramid, and other similar fibers.
- Finished products obtained from these materials.

## 3. Results

The results obtained with a frequency sweep of 10 to 300 Hz under an “input” of tensile stress-compression stress load with amplitudes of ±0.05 mm are shown in Table 2.

In the first column, the values of the frequencies were collected. Next, it was observed that there were two columns for each specimen. These columns provided us with information on dynamic stiffness and dynamic loss angle, respectively.

When analyzing the test results at ±0.05 mm (Table 2), it was observed that the two steel specimens, at equal thickness and volume and to Freq ≤ 110Hz, were much more rigid and uniform (around 34,000/mm) than two CFRTP material (between 11,729 N/mm y 12,923 N/mm).

It could be seen that for resonances greater than 100 Hz, the two steel specimens had a large dispersion in the values of the damping angle (Table 2). If we limited the resonance tests between 10 and 100 Hz, “the steel values” were more uniform (between 0.15° and 0.30°), but much lower than those obtained with the two “CFRTP test specimens” (between 2.26° and 1.21°).

To avoid resonance of the components with the steel, the test under a greater amplitude (±0.1mm) was performed with a lower frequency sweep (of 10 to 110 Hz). See the results in Table 3.

Analyzing the test results at ±0.1 mm amplitude (Table 3), it was observed that the stiffness in the two specimens of steel varied very little in relation to the 0.05 mm amplitude test and that, on the contrary, the dispersion of the damping angle improved considerably with values between 0.47° and 1.80°. Regarding the behavior of CFRTP test specimens, a similar behavior was observed in the stiffness, as well as an improvement in the damping angle (between 2.98° and 1.6°), which continued to be much higher than the angles observed with the two test specimens of steel.

It could be seen how, for a frequency of 110 Hz, the results were dispersed (the stiffness was reduced by half, and the angle was doubled), while in the CFRTP materials, both stiffness and angle hardly varied.

## 4. Discussion

Analyzing the results of Table 2 and Table 3, it could be seen that steel, at equal thickness and volume, was much more rigid than CFRTP material and that this was not significantly influenced by being subjected to different frequencies and amplitudes of resonance. The dynamic rigidity of steel was around 33,000 N/mm compared to 12,000 N/mm of the two CFRTPs, (Composite 1 and Composite 2).

According to Equation (1), given a constant force, greater damping (lower vibrations and rebound) is related to greater dynamic stiffness.

It could be seen that the CFRTP materials, even being much less rigid dynamically, had a much greater damping angle than steel.

**Figure 8:**
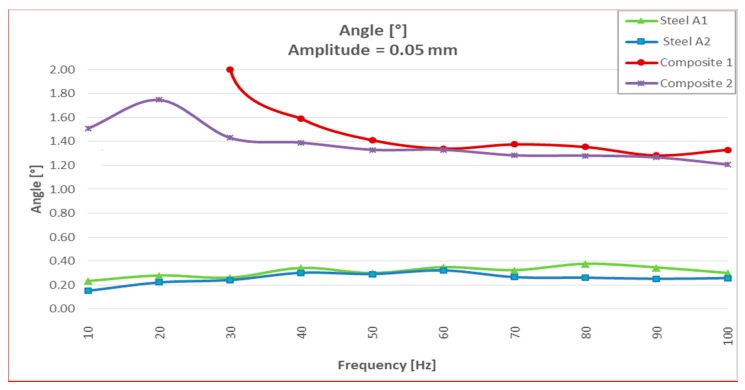
Dynamic loss angle results of CFRTP and steel, between 10 Hz and 100 Hz, at 0.5 mm.

Analyzing the values of the damping angle or dynamic loss, it was observed that the steel values between 10 and 100 Hz increased from three to five times by doubling the resonance amplitude (Figure 8 and Figure 14). However, the values obtained with CFRTP test specimens behaved uniformly and continued to be higher than the steel specimens.

In addition to these values, which demonstrated that CFRTP had better dynamic behavior than steel for frequencies below 100 Hz (Figure 8), the results over 100 Hz must also be analyzed.

**Figure 9:**
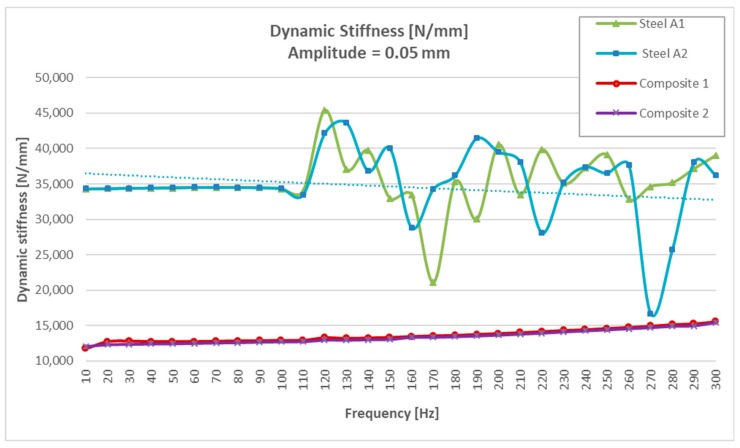
Dynamic stiffness results of CFRTP and steel, between 10 Hz and 300 Hz, at 0.05 mm.

**Figure 10:**
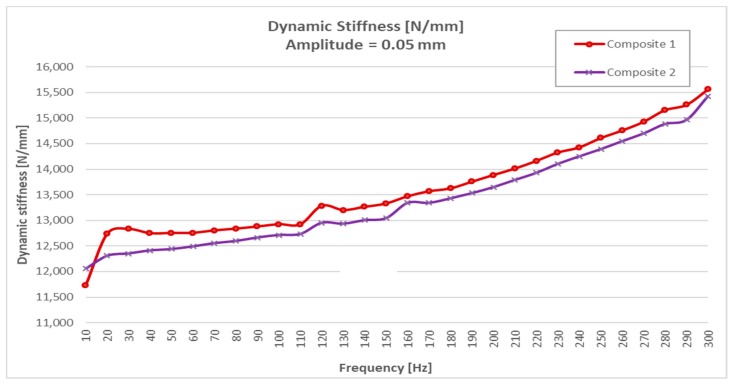
CFRTP dynamic stiffness results, between 10 Hz and 300 Hz, at 0.05 mm.

**Figure 11:**
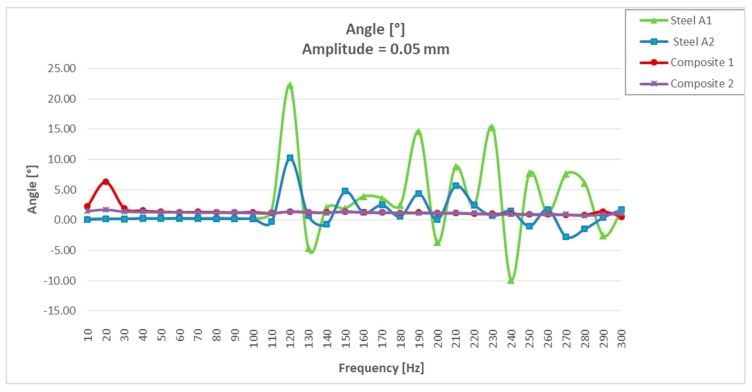
Dynamic loss angle results of CFRTP and steel, between 10 Hz and 300 Hz, at 0.05 mm.

**Figure 12:**
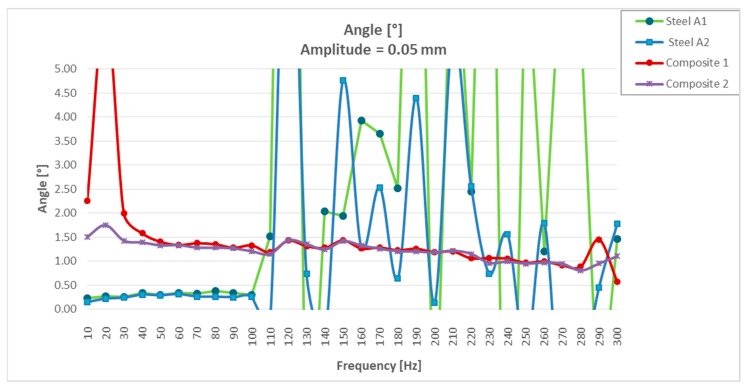
Dynamic loss angle results of CFRTP and steel, between 10 Hz and 300 Hz, expanded to 0°–5°.

Table 3 shows how there was a large dispersion of results in steel from this value. This means that it had entered resonance (Figure 8), which is very negative with respect to noise, because when a material enters resonance, the noise is very high, which translates it into a nuisance and lack of comfort for the driver and the passengers. However, in Figure 9, Figure 10, Figure 11 and Figure 12, it could be seen that the CFRTPs did not come into resonance under any circumstances.

**Figure 13:**
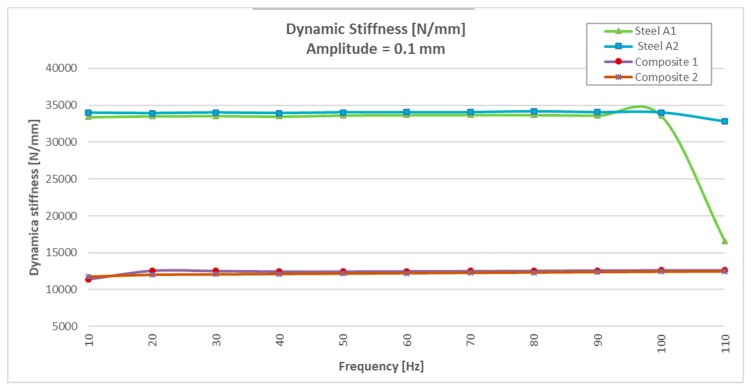
Dynamic stiffness results of CFRTP and steel, between 10 Hz and 110 Hz, at 0.1 mm.

The results obtained showed that even with 60% less dynamic stiffness (Figure 9 and Figure 13), CFRTPs had a damping capacity superior.

The stiffness/frequency results obtained in CFRTPs from 0 to 300 Hz area are shown in Figure 10.

The angle/frequency results obtained in CFRTP and steel test specimens from 0 to 300 Hz are shown in Figure 11. It was observed how the CFRTP remained stable throughout the working range, while the steel resonated from 100 Hz, producing uncontrolled undulations in the dynamic stiffness graphs, as well as in the dynamic angle graphs.

**Figure 14:**
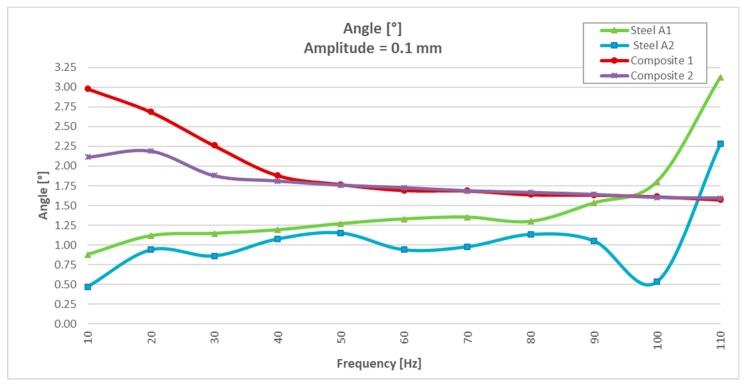
Dynamic loss angle results of CFRTP and steel, between 10 Hz and 110 Hz, at 0.1 mm.

This test was stopped at 110 Hz when it was observed that the steel specimens began to resonate, as in the previous test (Figure 13 and Figure 14).

The analysis of the values obtained in the dynamic test at 0.1 mm was the same for the amplitude of 0.05 mm:

- The stiffness of CFRTP reinforced was 60% lower than steel (Figure 13).
- The damping angle of CFRTP was greater than steel at any frequency and amplitude.
- The CFRTPs did not come into resonance under any circumstances.

### Future Investigations

A problem with CFRTP and any other plastic is mechanical bonding since these types of parts are always fixed to the chassis or some element of the suspension by means of mechanical joints.

**Figure 15:**
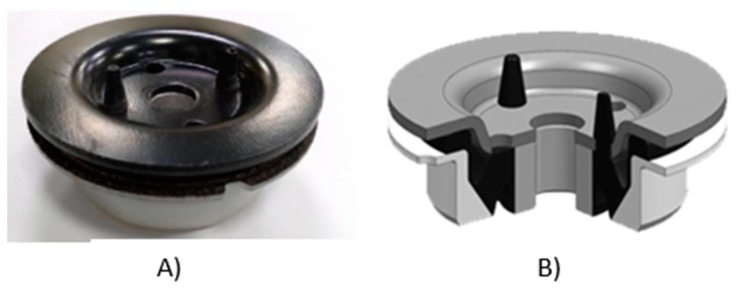
(**A**) Steel and vulcanized rubber gear mount, currently used as an anti-vibration car. (**B**) CAD3D model of CFRTP and vulcanized rubber.

To solve this problem, the authors have opened a parallel research line, (not included in this article), on the feasibility of CFRTP in fasteners, formed by an upper and a lower part of composite reinforced with continuous fiber, joined together by ultrasonic welding with an internal reinforcement between both sheets of the panel type. A component of the car, an anti-vibration differential gear assembly, made of steel and vulcanized rubber, is shown in Figure 15, as well as an experimental prototype made of CFRTP and vulcanized rubber.

**Figure 16:**
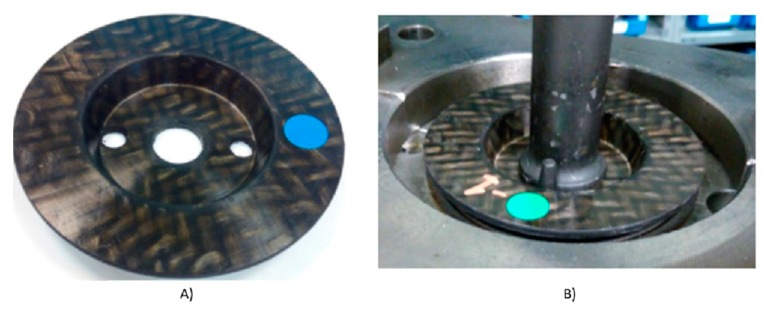
(**A**) Redesign of the CFRTP part. (**B**) CFRTP Part (PA 6 and P69) ready to begin the test.

In Figure 16, you could see the composite part made for thermoforming ready to be tested.

## 5. Conclusions

These new materials, CFRTP, offer coverage to every need of the automotive sector:

- These materials are more flexible than those currently used, such as steel or aluminum, allowing deformation in the event of a collision and absorbing more energy. In this way, the accelerations to which passengers are subjected are lower.
- They are more resistant to deformation, which makes it possible to avoid the penetration of any part of the vehicle into the security cabin, reducing the risk of injury to passengers. Thermoplastics have greater impact resistance than thermosets.
- These materials are lighter than the materials currently used. By reducing the weight, the power generated by the engine needed to move the vehicle will be lower, reducing fuel consumption and, thereby, reducing emissions of polluting gases into the atmosphere.
- Great capacity of absorption of the vibrations and noises is generated mainly by the engine, mechanical parts, wind, and disturbances of the road.
- High capacity of processing CFRTP materials offers very low production times as they can be processed automatically by thermoforming, unlike thermosetting matrix composites.
- Capability to be easily recycled, unlike their counterparts thermosetting composites, and have a greater advantage over steels.

It can be concluded that CFRTP materials are far superior to steels in dynamic, sound, and damping properties, making them ideal materials for the type of applications investigated.

## Figures and Tables

**Table 1:** Product range [Tepex ©].

Fiber	Polymer	Density [kg/dm^3^]	Fibre Content [vol.%]	Tensile Strength [MPa]	Tensile Modulus [GPa]	Flexural Strength [MPa]	Flexural Modulus [Pa]	Processing Temperat [°C]	Temperature in Use:	
									−Max Short Term [°C]	−Max Continuous [°C]
**Mechanical Properties**										
**Standard Materials**										
Roving Glass	PA66	1.8	45	472	23	600	21	280	200	130
Carbon	PA66	1.4	45	785	53	760	45	280	200	130
Roving Glass	PA6	1.8	45	405	22	620	19	240	180	120
Random Glass	PA6	1.6	35	195	13	260	12	240	180	120

**Table 2:** Dynamic test specimens’ results at ±0.05 mm.

AMPLITUDE 0.05 mm								
Freq.	STEEL A1		STEEL A2		COMPOSITE 1		COMPOSITE 2	
	Stiffness	Ang.	Stiffness	Ang.	Stiffness	Ang.	Stiffness	Ang.
Hz	[N/mm]	[°]	[N/mm]	[°]	[N/mm]	[°]	[N/mm]	[°]
10	34,256	0.233	34,331	0.147	11,729	2.262	12,055	1.506
20	34,342	0.279	34,361	0.219	12,741	6.288	12,307	1.751
30	34,360	0.261	34,427	0.24	12,830	1.998	12,352	1.427
40	34,418	0.341	34,452	0.302	12,747	1.586	12,411	1.390
50	34,410	0.299	34,498	0.29	12,752	1.407	12,438	1.330
60	34,479	0.347	34,538	0.314	12,755	1.339	12,490	1.333
70	34,534	0.323	34,539	0.264	12,803	1.376	12,554	1.283
80	34,507	0.375	34,531	0.259	12,837	1.354	12,596	1.281
90	34,478	0.345	34,478	0.250	12,878	1.283	12,664	1.266
100	34,304	0.299	34,359	0.256	12,923	1.331	12,713	1.206
110	33,994	1.514	33,487	−0.259	12,921	1.186	12,733	1.147
120	45,439	22.307	42,165	10.251	13,285	1.43	12,952	1.436
130	37,044	−4.653	43,656	0.734	13,201	1.316	12,934	1.355
140	39,726	2.035	36,846	−0.614	13,264	1.28	13,003	1.243
150	32,932	1.943	40,027	4.756	13,329	1.434	13,043	1.419
160	33,405	3.927	28,793	1.287	13,470	1.266	13,341	1.326
170	21,054	3.656	34,289	2.537	13,568	1.287	13,342	1.259
180	35,282	2.523	36,215	0.644	13,624	1.231	13,430	1.208
190	30,014	14.632	41,479	4.399	13,755	1.262	13,532	1.202
200	40,526	−3.663	39,515	0.130	13,886	1.189	13,649	1.183
210	33,485	8.844	38,101	5.692	14,011	1.206	13,790	1.218
220	39,846	2.455	28,101	2.563	14,161	1.064	13,929	1.154
230	35,077	15.351	35,187	0.74	14,326	1.068	14,104	0.962
240	37,237	−9.829	37,398	1.556	14,426	1.056	14,251	0.991
250	39,115	7.724	36,520	−0.963	14,608	0.969	14,392	0.948
260	32,850	1.209	37,678	1.795	14,756	0.997	14,548	0.969
270	34,637	7.642	16,703	−2.709	14,926	0.918	14,699	0.946
280	35,156	6.177	25,741	−1.459	15,154	0.89	14,885	0.804
290	37,167	−2.529	38,107	0.448	15,258	1.454	14,964	0.950
300	39,057	1.467	36,282	1.778	15,559	0.577	15,426	1.102

**Table 3:** Dynamic test specimens’ results at ±0.1 mm.

AMPLITUDE 0.1mm								
Freq.	STEEL A1		STEEL A2		COMPOSITE 1		COMPOSITE 2	
	Stiffness	Ang.	Stiffness	Ang.	Stiffness	Ang.	Stiffness	Ang.
Hz	[N/mm]	[°]	[N/mm]	[°]	[N/mm]	[°]	[N/m}	[°]
10	33,410	0.876	33,988	0.469	11,382	2.982	11,785	2.115
20	33,538	1.116	33,919	0.939	12,560	2.688	12,034	2.193
30	33,575	1.145	34,010	0.862	12,522	2.262	12,083	1.89
40	33,499	1.19	33,928	1.077	12,458	1.883	12,134	1.81
50	33,656	1.269	34,030	1.154	12,452	1.769	12,183	1.76
60	33,696	1.328	34,033	0.940	12,477	1.693	12,236	1.73
70	33,717	1.351	34,031	0.978	12,510	1.688	12,284	1.69
80	33,698	1.297	34,160	1.135	12,549	1.639	12,327	1.66
90	33,634	1.531	34,032	1.054	12,593	1.635	12,379	1.64
100	33,616	1.803	34,013	0.534	12,628	1.613	12,427	1.60
*110*	*16,623*	*3.126*	*32,798*	*2.281*	*12,629*	*1.574*	*12,459*	*1.59*
